# Effect of BMI on Mobility of Patients with Proximal Femoral Fracture

**DOI:** 10.5812/ircmj.2155

**Published:** 2013-09-05

**Authors:** Ali Yeganeh, Gholamreza Shah-Hoseini, Yaser Ghavami

**Affiliations:** 1 Orthopedic Department, RasoolAkram Hospital, Tehran, IR Iran; 2 General Practitioners, Tehran University of Medical Sciences, Tehran, IR Iran

**Keywords:** BMI, Mobility, Proximal Femoral Fracture

Dear Editor,

Proximal femoral fracture (PFF) is one of the most important etiologies of mortality and morbidity in elderly. Reported mortality rate is in a range of 1.3 - 16% pre-operatively up to 22 - 42% postoperatively (1, 2).Mortality rate in elderly patients with this problem is estimated about 30%.In Iran, and there are a relatively lower number of incidences for hip fracture than in western countries. Effective factors on mortality rate are aging, gender, intertrochanteric fractures and immobility before fracture (3). PFF usually requires a great amount of health care which causes patients to be hospitalized for a long time. Increased life expectancy leads to increased age and senescence. On the other hand, disorders such as cardiovascular diseases and diabetes result in additional concerns about complications caused by these fractures (4). Also increased rate of obesity in general population have worsened complications which have made treatments less effective (5). One of the most important therapeutic goals is to achieve movement ability after fracture in these patients. Walking with and without using crutch, and ability for mobility is expected in the cases (6). Established risk factors for decreased mobility in patients are osteoporosis, decreased bone density, prolonged consumption of corticosteroids, aging and central obesity. Several studies have shown direct relation between BMI and mortality rate in patients with the noted fractures (7, 8).

Primary surgical goal is retaining functional position after reduction of fracture and preventing avascular necrosis (AVN) of femoral neck, but it should be considered that reduction of mortality rate and movement capability is more important (9).

We studied records of patients who were hospitalized for PFF between March 2007 and March 2009 in Rasoul-Akram hospital (Tehran, Iran). According to BMI, patients were classified in four groups; including group 1 (BMI<20), group 2 (20≤ BMI < 25), group 3 (26≤BMI<29) and group 4 (BMI ≥ 30). Mobility of patients before and after fracture was compared. Three levels of movement were considered for these patients: complete movement, movement with crutch and immobility. Data were entered in a check list and analyzed using SPSS 16. Relationship between mobility (ability to move by feet) and BMI in these groups were calculated by Chi-square analysis. Among 94 patients, 51 (54.7%) were male and 43 (46%) were female. Average age of the patients was 48.4 ± 7.02 (youngest = 21, eldest = 85 year old). 16 patients had BMI less than 20, 35 patients had BMI between 20 - 25 (normal range) and 16 patients had BMI > 29.

Three different kinds of fractures were evaluated: femoral neck, intertrochanteric and sub trochanteric.

Hospitalization period was recorded for each patient. Initiation of therapy four days after fracture is considered as a therapeutic delay which was observed in 68 patients (30%). Average therapeutic delay was 5.5 ± 4 days (at least 1 day and at most 17 days). Mortality rate among these groups were compared to each other, and qualitative analysis revealed no significant correlation (Table 1).

**Table 1.** Comparison of Mobility Performed in Each of These FourGroups and Also Between Different Groups Mutually

	Before Treatment of PFF			After Treatment of PFF (15 months follow up)				
Group	Immobile (No.)	Using Crutch (No.)	Mobility Without Assistance (No.)	Disabled (No.)	Using Crutch (No.)	Mobility Without Assistanc e(No.)	Deceased (No.)	P value
**1**	6	5	5	8	3	2	3	0.035
**2**	5	3	27	3	1	25	7	0.076
**3**	4	4	18	3	2	13	9	0.082
**4**	6	4	6	4	3	3	6	0.024

In previous studies, there was less emphasis on BMI as a probable risk factor, and its effect on mobility after treatment was not well punctuated.It has been shown that mortality rate of patients with very low BMI in PFF is increased and this may be due to cardiovascular complications (10). In our study, results indicate that mobility before and after treatment of PFF are relevant to BMI. Mobility of patients with BMI < 20 and BMI > 30 was considerably decreased after treatment, i.e., mobility decreases in patients with BMI less or more than normal range. In other words, patients with morbid obesity or atrophic patients will suffer disability and decreased mobility after PFF. Moreover, comparison of these two groups with normal BMI patients (20<BMI<30), showed that mobility will not return to normal in morbid or atrophic patients.
